# Interrater Reliability of Chinese Medicine Diagnosis in People with Prediabetes

**DOI:** 10.1155/2013/710892

**Published:** 2013-05-09

**Authors:** Suzanne J. Grant, Rosa N. Schnyer, Dennis Hsu-Tung Chang, Paul Fahey, Alan Bensoussan

**Affiliations:** ^1^CompleMED, School of Science and Health, University of Western Sydney, Locked Bag 1797, Penrith, NSW 2751, Australia; ^2^School of Nursing, University of Texas, 1710 Red River Street, 5.188, Austin, TX 78701, USA; ^3^School of Science and Health, University of Western Sydney, Locked Bag 1797, Penrith, NSW 2751, Australia

## Abstract

*Background*. Achieving reproducibility in research design is challenging when patient cohorts under study are inconsistently defined. Traditional Chinese medicine (TCM) diagnosis is one example where inconsistency between practitioners has been found. We hypothesise that the use of a validated instrument may improve consistency. Biochemical biomarkers may also be used enhance reliability. *Methods*. Twenty-seven participants with prediabetes were assessed by two TCM practitioners using a validated instrument (TEAMSI-TCM). Inter-rater reliability was summarised using percentage agreement and the kappa coefficient. One-way ANOVA and Tukey's post hoc test were used to test links between TCM diagnosis and biomarkers. *Results*. The two practitioners agreed on primary diagnosis of 70% of participants. kappa = 0.56 (*P* < 0.001). The three predominant TCM diagnostic patterns for people with prediabetes were Yin deficiency, Qi and Yin deficiency and Spleen qi deficiency. The Spleen Qi deficiency with Damp cohort had statistically significant higher fasting glucose, higher insulin, higher insulin resistance, higher HbA1c and lower HDL than those with Qi and Yin deficiency. *Conclusions*. Using the TEAMSI-TCM resulted in moderate interrater reliability between TCM practitioners. This study provides initial evidence of variation in the biomarkers of people with prediabetes according to the different TCM patterns which may suggest a route to further improving interrater reliability.

## 1. Background 

INTERRATER reproducibility is regarded as one of the foundations of high quality research design. Achieving this standard is challenging when patient cohorts under study are poorly or inconsistently defined and in the absence of objective laboratory data [1, 2]. The subjective process of clinical diagnosis in traditional Chinese medicine (TCM) is one such example. Studies have demonstrated inconsistency between TCM practitioners in diagnosing the same patients [3–5]. We hypothesise, however, that it is possible to improve diagnostic consistency between practitioners, through the use of a validated instrument designed to reflect clinical practice and systematically guide practitioners. Once a TCM diagnosis is made, it may be possible to identify relationships between biochemical biomarkers and the diagnosis. This may be used to refine the instrument and improve reliability. 

In clinical practice, treatment of the patient depends on the differential diagnosis. Western medicine uses disease-based diagnosis, while TCM emphasizes patient-based diagnosis. In TCM, the patient's symptoms and signs are gathered through inquiry, observation, palpation, and smell. These symptoms and signs are interpreted into a diagnostic syndrome that often guides a patient specific (individualised) treatment [6, 7]. The same disease may have many different syndromes because of differences in symptoms and signs at different stages of the disease [8]. For example, two people presenting with the same (western) medical diagnosis of prediabetes may have a different clinical presentation leading to a different TCM diagnosis. One may be overweight with muscle aches and tiredness, while the other might have a wiry body, experience sweating on the hands and feet and a dry mouth. In TCM, these would be two different syndromes, Spleen Qi deficiency with Damp and Kidney Yin deficiency, respectively. The Chinese practitioner chooses or develops different herbal treatment formulas according to the patient's particular presentation and syndrome. Clinical research in TCM is made difficult by the use of this complex, individualised treatment [9].

For clinical research to be meaningful and usefully applied, it should reflect clinical practice as closely as possible. In order to best reflect clinical practice, TCM diagnostic principles should be incorporated into any clinical trial of TCM treatment [6, 7]. However, this is challenging, resource intensive and typically does not occur. Most research relies on applying a biomedical diagnosis and a standard herbal or acupuncture formula to all participants, with TCM diagnosis included in relatively few studies to date [10, 11]. Separating treatment from diagnosis may undervalue the efficacy of the TCM treatment being assessed [6, 12]. Methods which have been used to include a TCM diagnosis in clinical trials include the following:

1. allowing individualized patient treatment based on the TCM diagnosis [13, 14];
2. allocating patients to set treatment groups based on several overarching TCM diagnostic categories [15, 16];
3. assigning a fixed treatment with some capacity for additional herbs or acupuncture points based on TCM diagnosis [17].

Whilst these studies are commendable in that they aim to better reflect TCM practice to improve the relevance of the research, demonstrable interrater reliability of TCM diagnoses between practitioners is essential in order to ensure definitive, reproducible outcomes. If the diagnosis is unreliable, the appropriateness of the prescribed treatment may be in doubt.

Achieving a reliable diagnosis requires the use of standardized definitions and data collection methods. Methods of minimising variability in the diagnostic process include sharing the same patient history form so that all practitioners have the same information [18, 19] and using set practitioner questionnaires [4, 7]. Some studies have sought to reflect current clinical practice by allowing practitioners to use their own style of diagnostic assessment [20] or a minimal data collection and diagnosis form [21]. However, the lack of an adequate data collection instrument limits the precision with which a diagnosis can be consistently obtained by different practitioners and repeated. 

Poor process and poor definitions are likely to lead to variable results. Schnyer and colleagues have developed the Traditional East Asian Medicine Structured Interview, TCM version (TEAMSI-TCM) to assist the diagnostic process and minimise potential variability between individual practitioners presented with the same patient [12, 22]. 

Furthermore, it has been hypothesised that the reliability of TCM diagnosis may be enhanced by noting associations between patient clinical biomarkers and TCM diagnostic syndromes [23]. These relationships have increasingly been investigated in China [24, 25]. 

Our study aimed to (1) assess the interrater reliability of TCM diagnosis of people with prediabetes using a modification of TEAMSI-TCM and (2) document any relationships between biomarkers and TCM syndromes in people with prediabetes. 

## 2. Methods

### 2.1. Recruitment and Participants

Twenty-seven participants diagnosed with prediabetes by blood test were enrolled in the study. Men and women over 18 years of age with prediabetes were recruited in Sydney, Australia. Prediabetes is defined as having a fasting plasma glucose (FPG) level of <7.0 mmol/L and 2 hr plasma glucose load level ≥7.8 mmol/L and <11.0 mmol/L) This study was nested within a clinical trial investigating the effectiveness of Chinese herbal medicine in the treatment of impaired glucose tolerance (IGT) and insulin resistance in persons with prediabetes or mild diabetes. 

### 2.2. Practitioner Assessment

Two accredited TCM practitioners with 4-year Bachelor degrees in TCM from the University of Western Sydney, Australia, and more than 4-year clinical experience conducted the TCM diagnosis. Both practitioners were accredited with the relevant national professional association. The practitioners were both aware that the patient had been diagnosed with prediabetes. They were not required to provide a treatment plan. The practitioners were blind to each other's diagnosis until data entry was complete.

### 2.3. Diagnostic Instrument

The TEAMSI-TCM was used for data collection, modified to incorporate some syndromes specific to prediabetes. The instrument adopts a common TCM (Eight-Principle) approach to clinical differentiation. This is described more fully in standard texts [26]. 

The TEAMSI-TCM data collection instrument is designed to guide practitioners to use clinical signs, combine them in a systematic way, and generate a diagnosis. It is designed to be used in conjunction with education and training. It is structured in two parts—a patient questionnaire and a practitioner package [22].

A patient questionnaire was first completed by the participant. This provided self-reported data on the participant's symptoms. Practitioner 1 and Practitioner 2 read a copy of the same completed questionnaire prior to commencing the interview with the participant. Neither practitioner had access to the other's examination notes. To minimise measurement error, the interviews between patient and practitioner were usually conducted within an hour of each other in the same setting. The order in which a practitioner was seen varied according to appointment times and availability of practitioners. 

The practitioners' package consisted of a section for recording notes taken during the interview of the participant. Practitioners recorded general symptoms and symptoms related to the main complaint. An evaluation section provided guidance for recording observations relating to tongue, pulse, body, constitution, and complexion. 

The modification included a third form specifically designed for use in people with prediabetes. It allowed for a primary diagnostic syndrome or “pattern” and one or more secondary or accompanying patterns to be selected from a list. An option was provided for practitioners to contribute additional diagnoses or comments to modify the proposed diagnostic patterns provided on the form.

### 2.4. Diagnostic Criteria

A search of Chinese medicine journals and common texts identified eight distinct diagnostic patterns presented by people with prediabetes [27]. Nine diagnostic patterns were identified:

- Spleen deficiency with Damp (or Damp-heat),
- Qi and Yin deficiency,
- Qi deficiency,
- Yin deficiency (with Empty heat or Blood stasis),
- Phlegm-Damp Qi and Phlegm stagnation,
- Liver Qi stagnation (with Blood stasis),
- Yang deficiency,
- Blood stasis.

These patterns formed the basis of the diagnostic list of syndromes specific to prediabetes in the practitioner package. 

### 2.5. Statistical Analysis

To measure the level of agreement between practitioners on the primary and secondary syndromes selected, we adopted the approach used by Macpherson et al. [7]. We assessed the level of exact agreement on diagnosis and presented these as a percentage [28]. We also used the kappa coefficient as the main statistic to determine interrater reliability. Kappa is a measure of observed agreement between raters corrected for chance. A kappa of zero means the observed agreement is consistent with or less than chance agreement and a kappa of one means there is complete agreement [29]. MacPherson and colleagues recommended presenting both, the percentage of patients with congruent classifications and Cohen's kappa coefficient with 95% confidence limits. 

Possible associations between biomarkers and diagnostic patterns were investigated for those patients where both practitioners agreed on the primary diagnosis. As we sought to establish any links between the TCM diagnosis and the biomarkers, we preferred to use the diagnosis where reliability was clear. One-way ANOVA and Tukey's post hoc test were used to test for relationship between the diagnostic TCM patterns and each of the biomarker variables. In response to the modest sample size, we highlight all *P* values of less than 0.10 and interpret these as suggestive of potential relationships. Analyses were performed in SPSS version 18.0 (SPSS Inc., Chicago, IL), and CIs for values were calculated using the Wald approximation for 95% confidence intervals: estimate ±1.96 times the standard error.

## 3. Results

Twenty-seven participants with a mean age of 57.3 yrs (range 36–75 yrs) were recruited from June 2007 to December 2009. The mean fasting blood glucose was 6.2 mmol/L, and mean 2 hr OGTT was 10.6 mmol/L. The majority of participants had hypertension (*n* = 16) and/or high cholesterol (*n* = 14).

The two practitioners agreed exactly on 70% of the primary diagnoses for individual participants (see Table 1). Three TCM diagnostic patterns for people with prediabetes featured Yin deficiency, Qi and Yin deficiency, and Spleen Qi deficiency. These three patterns were fairly evenly distributed among the participants. The interrater reliability for the practitioners was found to be kappa = 0.56 (*P* < 0.001), 95% CI (0.25 to 0.81). This is statistically significantly higher than chance and represents a moderate level of agreement.

Secondary diagnostic patterns identified by practitioners were primarily Damp, Damp-heat, Liver Qi stagnation, and Blood stagnation (Table 2). Most participants (89%) received at least one secondary diagnosis by both practitioners. As multiple secondary patterns were identifiable for each participant, no attempt was made to calculate levels of agreement between practitioners. The hierarchical nature of secondary diagnosis within primary diagnoses was not amenable to kappa analysis. Exact agreement on both primary and secondary diagnostic patterns was 41% (Table 3).

An examination of the practitioners' TEAMSI-TCM notes from patient interviews and evaluations revealed some obvious reasons for different diagnoses. The main reasons appeared to be differences in what was divulged by a patient to one practitioner and not the other, differences in tongue and pulse evaluations, and varying levels of importance or weight according to certain symptoms. 

Where there was exact agreement on TCM diagnosis (*n* = 19), associations with biochemical markers were examined (Table 4). Despite the relatively small sample size, there was evidence that the Spleen Qi deficiency with Damp cohort had on average higher fasting glucose (*P* < 0.10), higher insulin (*P* < 0.05), higher insulin resistance (*P* < 0.05) than both those diagnosed with Qi and Yin deficiency and those participants with Yin deficiency alone. Participants with Spleen Qi deficiency and Damp also had a higher HbA1c (*P* < 0.10) than those participants diagnosed with Qi and Yin deficiency. Participants diagnosed with Spleen Qi deficiency and Damp were also distinct from those diagnosed with Yin deficiency, having higher triglycerides (*P* < 0.05), higher BMI (*P* < 0.05), and lower HDL (*P* < 0.10). There was no evidence of differences in mean biomarkers of people with Yin deficiency and Qi and Yin deficiency.

## 4. Discussion

This study aimed to assess the interrater reliability of traditional Chinese medicine diagnosis of people with prediabetes using a structured assessment instrument and to explore the relationship between TCM patterns and biomarkers of prediabetes. Although modest in size, it significantly contributes to an understanding of TCM diagnostic patterns in people with prediabetes. 

The analysis of 54 diagnoses of 27 participants found a moderate level of agreement between the two practitioners on the primary diagnosis. That is, the practitioners agreed on nearly 6 out of 10 diagnoses. The percentage of congruent classifications on the primary diagnosis was slightly higher at 70%. 

All but three participants in our study received at least one accompanying or secondary diagnosis. Diagnosis of multiple diagnostic patterns was a feature of previous research on interrater reliability [7, 30]. The diagnosis of secondary patterns tends to be subject to lower levels of interrater reliability [31]. This was reflected in our study where complete agreement between practitioners on both the primary and the accompanying diagnoses occurred in only 41% of cases.

The level of agreement on primary diagnosis found in our study is consistent with other interrater reliability studies where two practitioners were used [7, 20, 32]. It should be noted that in general when a diagnosis or clinical judgement requires a subjective assessment the interrater reliability between assessors is generally low [19]. This has been shown in studies assessing interrater reliability in diagnosing schizophrenia or assessing suspected stroke [33, 34]. 

The moderate level of agreement on the primary diagnosis achieved between the two practitioners in this study may be attributed to several factors—the similar backgrounds of the practitioners, the use of a thorough diagnostic instrument, and the limited number of practitioners involved. It is possible that a higher rate of agreement may have been reached if the diagnostic categories were constructed differently or training was conducted to ensure a shared understanding. 

Previous studies have found that differences in training affect the way a diagnosis is communicated [19, 21]. Our study limited this confounding factor by selecting practitioners who were trained in the same institution and with a similar length of practice experience. Conversely one study found that utilising TCM practitioners from a similar background and clinical experience made little or no difference to the average agreement of TCM diagnosis [19]. 

One of the strengths of this study was the use of the diagnostic TEAMSI-TCM tool. The benefits were manifold. The comprehensive patient questionnaire provided both practitioners with the same starting point. This ensured that a substantial amount of information was shared, and thereby limited variations of what was divulged in the patient-practitioner interview. Practitioners were guided to use a thorough assessment protocol rather than rely on pulse and/or tongue alone. A rationale for a diagnosis could clearly be seen through examining patient questionnaires and practitioner notes and evaluations. Our findings are supported by previous studies that have found that the use of more objective tools such as “inquiry” or questionnaire-based diagnoses process improves reliability [4, 35]. 

One of the most remarkable results of this study was that reliability of the TCM diagnostic framework was not null, given that prediabetes is a biomedical disorder determined by elevated blood glucose levels, without any overt symptoms and signs. Yet the TCM framework seems to provide an internally consistent framework to assess individual patient differences that may prove significant in prognosis or treatment. In TCM terms, these individuals with IGT are diagnostically “out of balance” in subtly different ways. The aim of TCM treatment is to readjust balance and enhance self-healing. Treatment is adjusted at each visit to meet the dynamic nature of the disease and prevent disease, in this case, progression to diabetes. The value of individualised treatment has been established in some studies [16, 36]. O'Brien et al. note that reproducibility may be higher in syndromes which are more commonly and better understood by practitioners [32]. 

No training was given on the diagnostic criteria for the patterns nominated in the TEAMSI-TCM tool. Different understanding and weighting of signs and symptoms of the three syndromes (Spleen Qi deficiency, Yin deficiency, and Qi and Yin deficiency) may have affected interrater reliability. There may have been improved interrater reliability if practitioners were taken through consensus training, or similar, to ensure that practitioners were all “speaking the same language” [35, 37].

Practitioner notes revealed factors which influence clinical judgement and are difficult to control. These are likely to be important barriers to reaching high levels of interrater reliability in TCM diagnosis. It would seem that the best approach is to utilise methods that guide diagnosis such as using a validated instrument. Even if we educate and train practitioners on these instruments and the nominated diagnostic categories, clinical judgement may confound the result to a lesser degree. Where the priority is to reach consensus for a clinical trial, a further strategy, suggested by O'Brien and Birch, may be used. Whereby two practitioners examine each patient and commence treatment only when diagnostic consensus has been reached [18].

Our results suggest that some differences in diagnosis were more likely than others. This implies some diagnostic patterns are more similar to each other than others. As further information accumulates about the relative “distances” between different diagnoses, weights for kappa may be developed to refine quantification of the magnitude of disagreement [38]. 

The three predominant TCM diagnostic patterns for people with prediabetes found in our study (Yin deficiency, Qi and Yin deficiency, and Spleen Qi deficiency) reflect those that had been found in previous literature. The pathophysiology of the progression from a prediabetic state of impaired glucose tolerance to diabetes can be seen in TCM as a progression from a deficiency of Spleen Qi with an ensuing stagnation of Damp, the generation of heat, consumption of fluids, and the emergence of Yin and Qi deficiency as the dominant pattern. Some patients will progress through this continuum, and different syndromes will dominate, while for others progression may not be so well defined [39–41]. In eight reviews of diagnostic patterns in people with prediabetes, spleen deficiency with Damp, and Qi and Yin deficiency, closely followed by Yin deficiency, were found to be dominant patterns. Blood stasis was a commonly identified secondary diagnostic pattern [24, 25, 39, 42–46]. 

The use of biomarkers integrated with TCM diagnosis has been proposed as a strategy that may improve reliability [23]. This small but emerging area of research has found relationships between certain diagnostic patterns and certain biomarkers. For example, eosinophils, which play an important role in the pathogenesis of bronchial asthma and allergic rhinitis, have been found to be more elevated in the patients with typical heat (*zheng*) [47]. Xu et al. (1993) found that high levels of platelet aggregation activity corresponded to blood stagnation [48]. Another study found that diagnosing blood stagnation in people with rheumatoid arthritis was possible through using a combination of proteomic and bioinformatics-based classification methods [49]. 

TCM diagnostic patterns for prediabetes are not well documented; nonetheless some research has been conducted on linking biomarkers to these patterns [24, 42]. In our research, we found that the Spleen Qi deficiency group had higher fasting blood glucose (FBG), insulin, greater insulin resistance, and higher HbA1c than the Qi and Yin deficiency group. This group also had higher FBG, insulin, BMI, and worse HDL cholesterol than the Yin deficiency group. The biomarker pathology for people with prediabetes diagnosed with Spleen Qi deficiency may therefore constitute a quite distinct group. This biochemical characterisation of Spleen Qi deficiency was similar to previous studies of biomarkers in prediabetes [24, 25, 50]. 

Chen and colleagues also found that people with prediabetes diagnosed with Qi and Yin deficiency tended to have hypertension and elevated low density lipids (LDL). We found that there was no significant variation in the biomarkers of people with Yin deficiency compared to those with Qi and Yin deficiency. This may have been because these groups were too similar or because the sample was too small.

Further research on the relationships between TCM diagnosis and biomarkers may provide further objective diagnostic criteria for use in clinical trials. 

As well as the small sample size and low statistical power, there are some other limitations to the design of this study. We used two practitioners to perform the diagnoses. Using a smaller number of raters was likely to result in higher percent agreement. Patients were diagnosed on one occasion only. This has been raised as a limitation in previous research, stating that patients are not generally seen only once by a practitioner in clinical practice [19, 37]. Typically a TCM diagnosis is refined over a few visits as signs and symptoms become more or less apparent through “inquiry,” “observation,” and “palpation.” There are several well-described limitations to the use of the kappa statistic that can influence the interpretation of results [51–53]. Very low (or high) prevalence results in high levels of expected agreement, and consequently the kappa value is often low despite near perfect agreement. We do not believe this occurred in our study. This study does not consider other diagnostic traditions of Chinese medicine. *Bianzheng lunzhi* or “determining treatment on the basis of discerning the syndrome” has achieved central position as the method for diagnosis in Chinese medicine. But in clinical practice this is one of many diagnostic and therapeutic strategies employed by clinicians [54].

## 5. Conclusion

When using the Eight-Principle Pattern Differentiation model characteristic of TCM, practitioners diagnosing patients with prediabetes commonly selected one of three patterns: Qi and Yin deficiency, Spleen Qi deficiency, or Yin deficiency. There was a moderate level of interrater reliability between practitioners. From the diagnostic data clear diagnostic patterns or TCM categories and subcategories of people with prediabetes are apparent. 

Our research contributes to the growing body of knowledge about the reliability of TCM diagnostic techniques and explores the potential use of biomarkers in enhancing this knowledge. Specifically it expands our understanding of the main TCM patterns present in people with prediabetes. 

The interrater reliability of TCM diagnosis merits wider study. This study has demonstrated a viable methodology. Future studies should include larger sample sizes of both practitioners and patients to address differences in training, education and clinical experience, conducting training, and “calibration” exercises. The larger samples sizes and accumulating knowledge of the relationship between diagnoses will inform the development of more refined statistical investigations (such as weights for the kappa statistic). The continued improvement of the face and content validity of the TEAMSI-TCM instrument will result in a robust tool for the conduct of interrater reliability studies and, in the future, clinical trials. 

## Figures and Tables

**Table 1:** Frequency of agreement of primary TCM diagnoses.

		Practitioner 1			
		Kidney Yin deficiency	Qi and Yin deficiency	Spleen Qi deficiency	Total
Practitioner 2	Kidney Yin deficiency	6 (22%)	1 (4%)	3 (11%)	10 (37%)
	Qi and Yin deficiency	0	7 (26%)	2 (7%)	9 (33%)
	Spleen Qi deficiency	0	2 (7%)	6 (22%)	8 (30%)

	Total	6 (22%)	10 (37%)	11	27 (100%)

**Table 2:** Secondary diagnostic syndrome only.

Accompanying syndrome	Practitioner	
	1	2
Damp heat	7	2
Spleen Qi deficiency	3	1
Plus Damp	11	14
Blood stagnation	5	9
Liver Qi stagnation	10	6
Yang xu	1	0
Phlegm	0	1
Yin deficiency	2	0

**Table 3:** Agreed primary and secondary diagnoses.

Pattern	*n*
Yin deficiency with spleen Qi deficiency	1
Yin deficiency with Damp heat	1
Yin deficiency with blood stagnation	1
Qi and Yin deficiency with Damp and blood stagnation	1
Qi and Yin deficiency with Damp	1
Spleen Qi deficiency with Damp and liver Qi stagnation	4
Spleen Qi deficiency with Damp heat and liver Qi stagnation	1
Spleen Qi deficiency with Damp heat	1

**Table 4:** TCM diagnosis for prediabetes and biomarkers.

Marker	TCM diagnosis			
	Yin deficiency (*n* = 6)	Qi and Yin deficiency (*n* = 7)	Spleen Qi deficiency with Damp (*n* = 6)	One-way ANOVA *P* value
Hypertension^*∧*^	3	5	3^a^	
Fasting blood glucose mmol/L (SD)	5.8 (0.8)	5.9 (0.7)	7.0 (0.9)*	0.054
2 hr glucose tolerance mmol/L (SD)	9.8 (2.2)	9.3 (1.6)	12.3 (3.4)	0.131
Insulin mmol/L (SD)	12.5 (8.3)	12.1 (4.6)	23.8 (8.6)**	0.027
Insulin resistance (SD)	1.8 (1.1)	1.6 (0.7)	3.2 (1.1)**	0.035
Glycated haemoglobin (HbA1c %) (SD)	6.1 (0.6)	6.0 (0.4)	6.6 (0.3)^†^	0.060
Triglycerides mmol/L (SD)	1.2 (0.6)	1.7 (0.7)	3.2 (2.2)^*∧∧*^	0.053
Total cholesterol mmol/L (SD)	4.8 (0.7)	5.1 (1.0)	4.8 (0.9)	0.755
High density lipoprotein mmol/L (SD)	1.8 (0.5)	1.6 (0.5)	1.1 (0.3)^*∧*^	0.074
Body Mass Index (SD)	27.7 (2.5)	28.7 (4.5)	33.7 (3.6)^*∧∧*^	0.057

^a^Biochemical data missing for one participant.

^*∧*^Refers to participants who were currently taking medication for hypertension.

*Difference between spleen Qi deficiency with Damp and the other two diagnostic categories (*P* < 0.10).

**Difference between spleen Qi deficiency with Damp and the other two diagnostic categories (*P* < 0.05).

^†^Difference between spleen Qi deficiency with Damp and Qi and Yin deficiency (*P* < 0.10).

^††^Difference between spleen Qi deficiency with Damp and Qi and Yin deficiency (*P* < 0.05).

^*∧*^Difference between spleen Qi deficiency with Damp and Yin deficiency (*P* < 0.10).

^*∧∧*^Difference between spleen Qi deficiency with Damp and Yin deficiency (*P* < 0.05).
